# Malaria Prophylaxis in Latin America: A Controversial Topic

**DOI:** 10.4269/ajtmh.2012.12-0193a

**Published:** 2012-07-01

**Authors:** Thomas Weitzel, Thierry Rolling, Gerd D. Burchard, Jakob P. Cramer

**Affiliations:** Travel Medicine Program; Clinica Alemana/Universidad del Desarrollo; Santiago, Chile; and; Institute of Tropical Medicine and; International Health Charité - University Medicine Berlin; Berlin, Germany; E-mail: thomas.weitzel@charite.de; I. Department of Internal Medicine; Section of Tropical Medicine; University Medical Center Hamburg-Eppendorf; Hamburg, Germany; I. Department of Internal Medicine; Section of Tropical Medicine; University Medical Center Hamburg-Eppendorf; Hamburg, Germany; and; Clinical Research Group; Bernhard Nocht Institute for Tropical Medicine; Hamburg, Germany

Dear Sir:

We read the comprehensive review of Steinhardt and colleagues on malaria prophylaxis for travelers to Latin America with great interest.^1^ Still, because this subject is controversial in Travel Medicine,^2^ we would like to add some information from Germany, a country not cited by the review.

Primaquine is the only available drug that eradicates hepatic stages of malaria parasites including hypnozoites of *Plasmodium vivax* and *Plasmodium ovale*. It is licensed for radical cure after vivax or ovale malaria.^3^ Only the U.S. American guidelines published by the Centers for Disease Control and Prevention (CDC) (www.cdc.gov) recommend it as a first-line option for primary prophylaxis. Therefore, the conclusion by Steinhardt and colleges to name primaquine as a recommended option for certain regions of Latin America is not based on a broad international consensus. To our personal experience, the use of this drug is hampered by several factors:

1. There is a lack of data and experience with primaquine in primary prophylaxis of malaria. Although the daily travel medicine practice deals mainly with brief regimens, most trials with primaquine have only evaluated longer regimens of over 12 weeks of prophylaxis.^4^ Furthermore, the capacity to prevent falciparum malaria, which is the main goal of malaria chemoprophylaxis^5^ has not been evaluated in larger studies. This is of importance, because in most Latin American areas with *P. vivax* predominance, *Plasmodium falciparum* co-occurs.^6^
2. Because the drug is not licensed or recommended for primary prophylaxis in any country except the United States, the prescribing physician is liable for any damage caused by primaquine. Furthermore, in many countries, the drug is not easily available as it has to be imported from foreign countries.
3. The necessary testing for glucose-6-phosphate-dehydrogenase (G6PD) activity before taking the drug adds significant costs and together with the delay of G6PD activity reporting renders the prophylactic use of primaquine impractical in daily travel medicine practice.

The incidence and mortality of autochthonous malaria in Latin America has declined dramatically since the early 2000s.^6^,^7^ During the years 2002–2010, six to 17 annual cases (average 13.0) of malaria imported from Latin American countries were notified to the German authorities (Robert Koch Institute, Berlin;www.rki.de). Absolute numbers were declining in recent years, but the relative share stayed stable with 2–4% of all imported malaria cases in Germany. For 2009, the German Federal Statistical Office reported more than 1.2 million German travelers to malaria-endemic countries in Latin America (www.destatis.de) resulting in six notified imported malaria cases from these countries (Brazil, 4; Honduras, 2), all caused by *P. vivax* (Schöneberg I, personal communication, Robert Koch Institute, Berlin). As a drawback to analysis, the proportion of German travelers to Latin America receiving chemoprophylaxis is unknown, but is expected to be very low in light of the German guidelines. The overall mortality rate for imported malaria in Germany in 2009 was 0.6% and is probably much lower for vivax malaria. Although the numbers might not be exact, they highlight the dimensions of the problem and confirm recent risk calculations based on endemicity data of malaria in the respective countries^8^: to prevent a single malaria case imported from Latin America to Germany, we have to use antimalarial drugs with their potentially severe side-effects in hundreds of thousands of travelers; to prevent a single fatal malaria case from Latin America in Germany, millions of travelers would have to use chemoprophylaxis. In Germany and Switzerland, chemoprophylaxis is only recommended for travelers to certain areas of Brazil (Acre, Rondónia, and Roraima) and all non-coastal regions in Guyana, Suriname, and French Guiana (www.dtg.org, www.safetravel.ch). For all other parts of Latin America, avoidance of mosquito bites and immediate consultation of a physician in case of fever during or after the journey is recommended. Travelers, who stay in areas where the diagnosis and treatment of malaria might not be possible in a timely manner, carry a drug for self-treatment (“stand-by emergency treatment”). This approach aims to avoid the broad use of chemoprophylaxis in areas of low malaria risk and therefore, from a medical and health economical point of view, might be a preferable option for tourists to many Latin America regions.
